# Supplementary material for: Understanding public health risk from unsafe dry fish consumption in Bangladesh
Source: PLoS One. 2024 Nov 13;19(11):e0310826. doi: 10.1371/journal.pone.0310826 (PMC11560022; doi:10.1371/journal.pone.0310826)
Supplement: S1 Table — (DOCX) [file pone.0310826.s001.docx]

**S1 Table.** Gender-wise knowledge regarding health hazard due to dried fish consumption (N=415, November-December 2022, Bangladesh).

| Characteristics | Total  n (%) | Male  n (%) | Female  n (%) | *p*-value |
| --- | --- | --- | --- | --- |
| Do you think consuming preservative free dried fish is good for health? | | | | |
| Yes | 154 (37.1) | 81 (19.5) | 73 (17.6) | 0.137 |
| No | 121 (29.2) | 72 (17.3) | 49 (11.8) |  |
| Don’t know | 140 (33.7) | 66 (15.9) | 74 (17.8) |  |
| Do you think that dried fish is a good source of protein? | | | | |
| Yes | 200 (48.2) | 106 (25.5) | 94 (22.7) | 0.391 |
| No | 97 (23.4) | 46 (11.1) | 51 (12.3) |  |
| Don’t know | 118 (28.4) | 67 (16.1) | 51 (12.3) |  |
| Do you know about the methods (natural and mechanical) of fish drying? | | | | |
| Yes | 214 (51.6) | 121 (29.2) | 93 (22.4) | 0.166 |
| No | 156 (37.6) | 73 (17.6) | 83 (20.0) |  |
| Don’t know | 45 (10.8) | 25 (6.0) | 20 (4.8) |  |
| Do you know the use of illegal pesticides in dried fish? | | | | |
| Yes | 148 (35.7) | 83 (20.0) | 65 (15.7) | 0.363 |
| No | 153 (36.9) | 82 (19.8) | 71 (17.1) |  |
| Don’t know | 114 (27.5) | 54 (13.0) | 60 (14.5) |  |
| Do you know about the presence of heavy metals (Pb, Cd, Cr, Hg, As, etc.) in dried fish? | | | | |
| Yes | 135 (32.5) | 77 (18.6) | 58 (14.0) | 0.480 |
| No | 157 (37.8) | 80 (19.3) | 77 (18.6) |  |
| Don’t know | 123 (29.6) | 62 (14.9) | 61 (14.7) |  |
| Do you know about the presence of hydroperoxides and free radicals in dried fish? | | | | |
| Yes | 59 (14.2) | 37 (8.9) | 22 (5.3) | 0.239 |
| No | 202 (48.7) | 105 (25.3) | 97 (23.4) |  |
| Don’t know | 154 (37.1) | 77 (18.6) | 77 (18.6) |  |
| Do you know about the presence of microplastics in dried fish? | | | | |
| Yes | 97 (23.4) | 57 (13.7) | 40 (9.6) | 0.302 |
| No | 176 (42.4) | 93 (22.4) | 83 (20.0) |  |
| Don’t know | 142 (34.2) | 69 (16.6) | 73 (17.6) |  |
| Do you know about the presence of pathogenic microbial contamination in dried fish? | | | | |
| Yes | 151 (36.4) | 83 (20.0) | 68 (16.4) | 0.678 |
| No | 147 (35.4) | 78 (18.8) | 69 (16.6) |  |
| Don’t know | 117 (28.2) | 58 (14.0) | 59 (14.2) |  |
| Do you know hazard-contaminated fishes can have same variations in color, texture, odor or taste? | | | | |
| Yes | 119 (28.7) | 63 (15.2) | 56 (13.5) | 0.728 |
| No | 179 (43.1) | 91 (21.9) | 88 (21.2) |  |
| Don’t know | 117 (28.2) | 65 (15.7) | 52 (12.5) |  |
| Do you know the health hazard associated with contaminated dried fish consumption (i.e., cancer due to DDT)? | | | | |
| Yes | 174 (41.9) | 93 (22.4) | 81 (19.5) | 0.094 |
| No | 143 (34.5) | 83 (20.0) | 60 (14.5) |  |
| Don’t know | 98 (23.6) | 43 (10.4) | 55 (13.3) |  |
| Do you know the storage process for dried fish? | | | | |
| Yes | 177 (42.7) | 98 (23.6) | 79 (19.0) | 0.557 |
| No | 175 (42.2) | 87 (21.0) | 88 (21.2) |  |
| Don’t know | 63 (15.2) | 34 (8.2) | 29 (7.0) |  |
| Do you know the storage hazards (i.e., spoilage, beetles, mites, etc.) in dried fish? | | | | |
| Yes | 245 (59.0) | 126 (30.4) | 119 (28.7) | 0.646 |
| No | 108 (26.0) | 57 (13.7) | 51 (12.3) |  |
| Don’t know | 62 (14.9) | 36 (8.7) | 26 (6.3) |  |
| Do you know the average shelf life of naturally stored dried fish? | | | | |
| Yes | 77 (18.6) | 41 (9.9) | 36 (8.7) | 0.892 |
| No | 224 (54.0) | 120 (28.9) | 104 (25.0) |  |
| Don’t know | 114 (27.5) | 58 (14.0) | 56 (13.5) |  |
