# Supplementary material for: Understanding public health risk from unsafe dry fish consumption in Bangladesh
Source: PLoS One. 2024 Nov 13;19(11):e0310826. doi: 10.1371/journal.pone.0310826 (PMC11560022; doi:10.1371/journal.pone.0310826)
Supplement: S2 Table — (DOCX) [file pone.0310826.s002.docx]

**S2 Table.** Gender-wise attitudes toward health hazard due to dried fish consumption (N=415, November-December 2022, Bangladesh).

| Characteristics | Total  n (%) | Male  n (%) | Female  n (%) | *p*-value |
| --- | --- | --- | --- | --- |
| Majority of the country’s people do not eat dry fish because of its smell and taste | | | | |
| Agree | 333 (80.2) | 171 (41.2) | 162 (39.0) | 0.472 |
| Disagree | 56 (13.5) | 32 (7.7) | 24 (5.8) |  |
| Undecided | 26 (6.3) | 16 (3.9) | 10 (2.4) |  |
| Consumers should have some knowledge on the hazards of dried fish before buying | | | | |
| Agree | 387 (93.3) | 202 (48.7) | 185 (44.6) | 0.637 |
| Disagree | 14 (3.4) | 8 (1.9) | 6 (1.5) |  |
| Undecided | 14 (3.4) | 9 (2.2) | 5 (1.2) |  |
| Buyers should ask questions about the use of chemicals (pesticides, insecticides, etc.) in dried fish to seller | | | | |
| Agree | 366 (88.2) | 190 (45.8) | 176 (42.4) | 0.060 |
| Disagree | 24 (5.8) | 18 (4.3) | 6 (1.4) |  |
| Undecided | 25 (6.0) | 11 (2.7) | 14 (3.4) |  |
| Color of dried fish is an indicator of chemical contamination | | | | |
| Agree | 101 (24.3) | 53 (12.8) | 48 (11.6) | 0.800 |
| Disagree | 142 (34.2) | 78 (18.8) | 64 (15.4) |  |
| Undecided | 172 (41.4) | 88 (21.2) | 84 (20.2) |  |
| The price of chemical free dried fish is comparatively higher | | | | |
| Agree | 268 (64.6) | 147 (35.4) | 121 (29.2) | 0.514 |
| Disagree | 34 (8.2) | 17 (4.1) | 17 (4.1) |  |
| Undecided | 113 (27.2) | 55 (13.3) | 58 (14.0) |  |
| Consumers should check ectoparasitic infestation (fleas, flies, mites, etc.) before buying | | | | |
| Agree | 393 (94.7) | 207 (49.9) | 186 (44.8) | 0.654 |
| Disagree | 4 (1.0) | 3 (0.7) | 1 (0.2) |  |
| Undecided | 18 (4.3) | 9 (2.2) | 9 (2.2) |  |
| Final product should come in an airtight polythene pouch or closed container | | | | |
| Agree | 338 (81.4) | 188 (45.3) | 150 (36.1) | 0.050 |
| Disagree | 14 (3.4) | 6 (1.4) | 8 (1.9) |  |
| Undecided | 63 (15.2) | 25 (6.0) | 38 (9.2) |  |
| Dried fish should sometimes be sun-dried when stored at home | | | | |
| Agree | 299 (72.0) | 154 (37.1) | 145 (34.9) | 0.709 |
| Disagree | 32 (7.7) | 18 (4.3) | 14 (3.4) |  |
| Undecided | 84 (20.2) | 47 (11.3) | 37 (8.9) |  |
| Washing with warm water before cooking can reduce the level of chemicals | | | | |
| Agree | 328 (79.0) | 168 (40.5) | 160 (38.6) | 0.088 |
| Disagree | 18 (4.3) | 14 (3.4) | 4 (1.0) |  |
| Undecided | 69 (16.6) | 37 (8.9) | 32 (7.7) |  |
| Cooking destroys all the hazards | | | | |
| Agree | 141 (34.0) | 79 (19.0) | 62 (14.9) | 0.580 |
| Disagree | 116 (28.0) | 61 (14.7) | 55 (13.3) |  |
| Undecided | 158 (38.1) | 79 (19.0) | 79 (19.0) |  |
| If you were made aware of the presence of heavy metals in dried fish, you would change your buying or consuming habit | | | | |
| Agree | 329 (79.3) | 177 (42.7) | 152 (36.6) | 0.592 |
| Disagree | 30 (7.2) | 16 (3.9) | 14 (3.4) |  |
| Undecided | 56 (13.5) | 26 (6.3) | 30 (7.2) |  |
| If you were made aware that cancer can be caused by consuming chemically contaminated dried fish, you would change your consumption habit | | | | |
| Agree | 348 (83.9) | 185 (44.6) | 163 (39.3) | 0.751 |
| Disagree | 25 (6.0) | 14 (3.4) | 11 (2.7) |  |
| Undecided | 42 (10.1) | 20 (4.8) | 22 (5.3) |  |
| Dried fish packaging must include production and expiry date | | | | |
| Agree | 382 (92.0) | 200 (48.2) | 182 (43.9) | 0.069 |
| Disagree | 12 (2.9) | 10 (2.4) | 2 (0.5) |  |
| Undecided | 21 (5.1) | 9 (2.2) | 12 (2.9) |  |
| Dried fish should be certified by health inspectors | | | | |
| Agree | 389 (93.7) | 207 (49.9) | 182 (43.9) | 0.747 |
| Disagree | 10 (2.4) | 5 (1.2) | 5 (1.2) |  |
| Undecided | 16 (3.9) | 7 (1.7) | 9 (2.2) |  |
| You will participate in consumer awareness program on health hazards of dried fish | | | | |
| Agree | 281 (67.7) | 151 (36.4) | 130 (31.3) | 0.021 |
| Disagree | 22 (5.3) | 17 (4.1) | 5 (1.2) |  |
| Undecided | 112 (27.0) | 51 (12.3) | 61 (14.7) |  |
