# Supplementary material for: Understanding public health risk from unsafe dry fish consumption in Bangladesh
Source: PLoS One. 2024 Nov 13;19(11):e0310826. doi: 10.1371/journal.pone.0310826 (PMC11560022; doi:10.1371/journal.pone.0310826)
Supplement: S3 Table — (DOCX) [file pone.0310826.s003.docx]

**S3 Table.** Gender-wise practices of dried fish consumption among the studied population (N=415, November-December 2022, Bangladesh).

| Characteristics | Total  n (%) | Male  n (%) | Female  n (%) | *p*-value |
| --- | --- | --- | --- | --- |
| Do you buy dried fish from a trusted seller/processor? | | | | |
| Yes | 143 (34.5) | 78 (18.8) | 65 (15.7) | 0.807 |
| No | 192 (46.3) | 101 (24.3) | 91 (21.9) |  |
| Sometimes | 80 (19.3) | 40 (9.6) | 40 (9.6) |  |
| Do you consider the price of dried fish instead of quality while purchasing? | | | | |
| Yes | 100 924.1) | 50 (12.1) | 50 (12.1) | 0.090 |
| No | 228 (54.9) | 114 (27.5) | 114 (27.5) |  |
| Sometimes | 87 (21.0) | 55 (13.3) | 32 (7.7) |  |
| Do you verify the presence of hazards before buying by asking sellers? | | | | |
| Yes | 129 (31.1) | 69 (16.6) | 60 (14.5) | 0.509 |
| No | 234 (56.4) | 119 (28.7) | 115 (27.7) |  |
| Sometimes | 52 (12.5) | 31 (7.5) | 21 (5.1) |  |
| Do you buy dried fishes which are free of flies, insects, or rodents? | | | | |
| Yes | 259 (62.4) | 126 (30.4) | 133 (32.1) | 0.008 |
| No | 134 (32.3) | 75 (18.1) | 59 (14.2) |  |
| Sometimes | 22 (5.3) | 18 (4.3) | 4 (1.0) |  |
| Do you buy dried fishes that are free of microbial spoilage? | | | | |
| Yes | 309 (74.5) | 159 (38.3) | 150 (36.1) | 0.578 |
| No | 78 (18.8) | 43 (10.4) | 35 (8.4) |  |
| Sometimes | 28 (6.7) | 17 (4.1) | 11 (2.7) |  |
| Do you ask the seller about drying technique and choose mechanically dried fishes over sun dried fishes? | | | | |
| Yes | 75 (18.1) | 42 (10.1) | 33 (8.0) | 0.820 |
| No | 264 (63.6) | 137 (33.0) | 127 (30.6) |  |
| Sometimes | 76 (18.3) | 40 (9.6) | 36 (8.7) |  |
| Do you ask the shelf life of dried fish before buying? | | | | |
| Yes | 110 (26.5) | 57 (13.7) | 53 (12.8) | 0.953 |
| No | 251 (60.5) | 134 (32.3) | 117 (28.2) |  |
| Sometimes | 54 (13.0) | 28 (6.8) | 26 (6.3) |  |
| Do you store dried fish at home after airtight packaging? | | | | |
| Yes | 229 (55.2) | 106 (25.5) | 123 (29.6) | 0.012 |
| No | 139 (33.5) | 86 (20.7) | 53 (12.8) |  |
| Sometimes | 47 (11.3) | 27 (6.5) | 20 (4.8) |  |
| Do you preserve dried fish in a dry and clean place? | | | | |
| Yes | 322 (77.6) | 163 (39.3) | 159 (38.3) | 0.225 |
| No | 57 (13.7) | 33 (8.0) | 24 (5.8) |  |
| Sometimes | 36 (8.7) | 23 (5.5) | 13 (3.1) |  |
| To prevent moisture reabsorption, do you occasionally re-dry fishes in the sun that are stored at home? | | | | |
| Yes | 185 (44.6) | 98 (23.6) | 87 (21.0) | 0.440 |
| No | 155 (37.3) | 77 (18.6) | 78 (18.8) |  |
| Sometimes | 75 (18.1) | 44 (10.6) | 31 (7.5) |  |
| Do you wash dried fishes with hot water before cooking? | | | | |
| Yes | 334 (80.5) | 173 (41.7) | 161 (38.8) | 0.672 |
| No | 42 (10.1) | 23 (5.5) | 19 (4.6) |  |
| Sometimes | 39 (9.4) | 23 (5.5) | 16 (3.9) |  |
