# Supplementary material for: Understanding public health risk from unsafe dry fish consumption in Bangladesh
Source: PLoS One. 2024 Nov 13;19(11):e0310826. doi: 10.1371/journal.pone.0310826 (PMC11560022; doi:10.1371/journal.pone.0310826)
Supplement: Attachment — Submitted filename: Plos one review.docx [file pone.0310826.s005.docx]

1. Keep keywords in alphabetical order in abstract.
2. In line number66, “There have been no research studies on Bangladesh's food safety problems resulting from the consumption of unsafe dry fish.” How can you say that no research studies yourself?
3. How was the sample size calculated in your study? Explain properly.
